# The complete chloroplast genome of *Salix psamaphila*, a desert shrub in northwest China

**DOI:** 10.1080/23802359.2019.1675485

**Published:** 2019-10-09

**Authors:** Dongye Lu, Lei Hao, Haiguang Huang, Guosheng Zhang

**Affiliations:** a College of Forestry, Inner Mongolia Agricultural University, Hohhot, China;; b College of Resources and Environmental Economics, Inner Mongolia University of Finance and Economics, Hohhot, China;; c Inner Mongolia Academy of Forestry Science, Hohhot, China

**Keywords:** Chloroplast genome, *Salix psammophila* C. Wang & Chang Y. Yang, Salicaceae, phylogenetic analysis

## Abstract

*Salix psammophila* C. Wang & Chang Y. Yang is a desert plant species distributed in Northwest China. Here, we report the complete choroplast genome sequences in order to enrich its genetic resource. The total genome is 155,278 bp in length and contains a typical quadripartite structure, including a large single copy (LSC) region of 84,457 bp, a small single copy (SSC) region of 15,891 bp, and a pair of inverted repeats (IRs) of 27,465 bp. There were 130 genes in the genome, including 85 protein-coding genes, 37 tRNA genes and eight rRNA genes. The overall CG content in the plastome of *S. psammophila* is 36.72%. The phylogenetic tree based on 18 complete plastomes of Salicaceae support close relationships among *S. psammophila*, *Salix taoensis*, and *Salix rehderiana*.

The Salicaceae species, which grow rapidly and easy to asexually propagate, are known for greatly ecological and economic value as well as ideal research objects for genetics and forestry breeding. *Salix psammophila* C. Wang et Chang Y. Yang, which is an important wind-breaking and sand-fixing tree species with rich phenotypic traits in the Northwest China (China 2013), but its chloroplast genetic resources and phylogenetic status is still unknown. Therefore, in order to better develop, utilise, manage and protect the *S. psammophila* germplasm resources, the complete chloroplast genome was finished using high-throughput Illumina pair-end sequencing technology and the phylogeny of *Salix* species was constructed based on the reported complete chloroplast genome sequences.

The fresh leaves were collected from Inner Mongolia Forest Tree Breeding Centre (Tumd Left Banner, Hohhot, China; Coordinates: 111.722E, 40.5646 N; Altitude: 1063 m). The voucher specimens of *S. psammophila* (SPS0502) were deposited at the herbarium of Inner Mongolia Agriculture University. Total genome DNA was extracted using a Plant Genomic DNA Kit (TIANGEN, Beijing, China). The chloroplast genome sequencing was performed using the Illumina HiSeq2500 Platform (Illumina, CA, USA). In total, about 6.0 GB clean reads were assembled with the reference cpDNA sequence of *Salix rehderiana* (MG262367.1) using the software Bowtie v2.3.5 (Langmead and Salzberg 2012). All of the plastid-like reads were assembled into contigs by SPAdes v3.10.1 (Bankevich et al. 2012). The chloroplast genome of *S. psammophila* was annotated using the DOGMA web server (Wyman et al. 2004) but Initial annotation, putative starts, stops and intron positions were determined by comparison with homologus genes in other cp genomes. A physical map of the circular genome was generated with OGDRAW v1.2 (Lohse et al. 2013). The annotated genome sequence has been deposited to GenBank under the Accession Number MN495627.

The circular cp genome of *S. psammophila* was 155,278 bp in size, containing a large single copy (LSC) region of 84,457 bp, a small single copy (SSC) region of 15,891 bp, and a pair of inverted repeats of 27,465 bp. There were 130 genes in the genome, of which 85 are protein-coding genes (PCGs), 37 are tRNA genes and eight are ribosomal RNA genes. Most of the genes occur as a single copy, and 18 genes are duplicated in IR regions. The overall CG content of the cp genome is 36.72%, while the corresponding values of the LSC, SSC, and IR regions are 34.44%, 31.07%, and 41.86%, respectively.

With the plastomes of *Idesia polycarpa* and *Itoa orientalis* (Flacourtiaceae) as outgroups, We used RAxML v8.2.10 (Stamatakis 2014) with 1000 bootstraps under the GTRGAMMAL substitution model to constrtuct a maximum likelihood(ML) phylogeny of 18 species of the genus Salicaceae in the NCBI database, which were aligned by the MAFFT v7.037 (Katoh and Standley 2013). The phylogenetic analysis suggested that *S. psammophila* is closely related to the species of *Salix taoensis* and *Salix rehderiana* (Figure 1). This sequence of the chloroplast genome will be of use for the conservation of genetic resource for *S. psammophila* as well as the phylogenetic evolution of Salicaceae.

**Figure 1.**
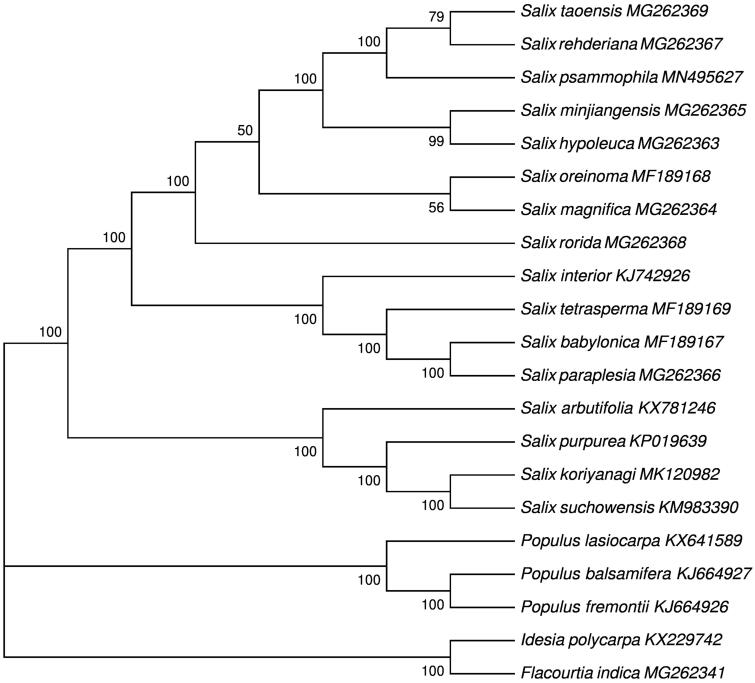
The ML phylogenetic tree for *S. psammophila* based on 18 chloroplast genome sequences of Salicaceae and two plastomes of Flacourtiaceae were selected as outgroups.
